# Evaluating quality improvement in tertiary care hospital before and after NABH accreditation: a systematic review

**DOI:** 10.3389/frhs.2025.1654514

**Published:** 2025-08-26

**Authors:** Deepika Kanyal, Babaji Ghewade

**Affiliations:** Department of Hospital Administration, Datta Meghe Institute of Medical Sciences, Wardha, India

**Keywords:** NABH, quality indicators, patient satisfaction, healthcare standards, hospital-acquired infections, accreditation, risk of bias

## Abstract

**Background:**

The National Accreditation Board for Hospitals and Healthcare Providers (NABH.) accreditation process aims to elevate the quality of healthcare services through an impartial, external peer evaluation of an organization's performance. This study compares NABH. quality indicators in a tertiary care hospital before and after accreditation, assessing changes in patient care, hospital management, and staff performance.

**Methods:**

A systematic review was conducted following PRISMA guidelines. Literature was searched across PubMed, Scopus, Google Scholar, and the Cochrane Library from August 2023 to January 2024. Keywords included “NABH. accreditation and hospital quality,” “Accreditation in Indian hospitals and patient safety,” and “Tertiary care hospital before and after accreditation.” Boolean operators, synonyms, and related terms were used to ensure comprehensive retrieval. Independent reviewers screened studies, and risk of bias assessment was performed using the Cochrane Risk of Bias Tool.

**Results:**

The study highlights significant improvements in hospital quality indicators following NABH accreditation. Hospital-acquired infection rates decreased, with infection control compliance improving by 40%. Operational efficiency improved with a 20% reduction in discharge delays and a 15% increase in documentation accuracy. Patient satisfaction scores rose by 25%, and structured policies enhanced service quality by 30%. Additionally, 85% of hospital staff reported higher job satisfaction. Statistical analysis confirmed significant differences in compliance rates (*p* < 0.05) and patient care metrics (*p* < 0.01). Despite initial implementation challenges due to resource constraints, the study underscores the need for continuous monitoring and reinforcement of accreditation standards to sustain these improvements.

**Conclusion:**

NABH accreditation improves patient safety, lowers infection rates, and boosts overall productivity. The structured framework encourages continuous improvement, but institutional commitment and ongoing oversight are necessary for long-term sustainability.

## Introduction

Quality in healthcare defined as the degree to which health services increase the likelihood of desired health outcomes and are consistent with current professional knowledge. Globally, health systems have increasingly adopted quality improvement initiatives to achieve measurable enhancements in clinical care and patient satisfaction.

In order to provide high-quality healthcare services in India, hospitals must be accredited by the National Accreditation Board for Hospitals and Healthcare Providers (NABH.). The thorough and exacting process of NABH. accreditation is intended to guarantee that healthcare institutions adhere to set criteria for patient care, safety, and organizational management. NABH. aligns its standards with international best practices, particularly those of the International Society for Quality in Healthcare (ISQua), as a constituent board of the Quality Council of India (Q.C.I.) (1). A healthcare institution must undergo frequent evaluations in order to receive NABH. accreditation, which encourages advancements in the operational and structural areas of healthcare delivery (2).

To ensure that hospitals meet and continuously strive to exceed predefined benchmarks in important areas like patient safety, clinical care, infection control, and patient rights, the accreditation process entails an external evaluation by a qualified team of healthcare professionals who look at the organization's policies, procedures, and outcomes (3). The evaluation includes a self-assessment by the hospital, followed by an on-site survey where the external team reviews hospital operations and interacts with staff and patients. A report is then compiled that may include recommendations for improvement, and the hospital's status is then decided—either it is granted accreditation or further changes are necessary (4–6).

Accreditation contributes to the standardization of care delivery procedures, which lowers errors and fosters a culture of quality and safety (7).

Although NABH. accreditation has several advantages, the procedure is time-consuming and labor-intensive. Hospitals may find it challenging to achieve strict accreditation requirements, especially when it comes to infrastructure upgrades, personnel training, and budget allocation. Accreditation preparation can be a significant initial commitment that takes time and money. However, the long-term advantages—such as better patient care, more operational effectiveness, and a higher reputation in the healthcare sector—often exceed these difficulties (8).

This study compares performance metrics before and after certification in order to evaluate the effect of NABH. accreditation on quality indicators within a tertiary care hospital. Through an analysis of the process's observable advantages and difficulties, the research will provide significant additional information about how accreditation can promote quality enhancement in healthcare institutions (9).

PICOS Framework:

• **Population (P):** Indian tertiary care hospitals
• **Intervention (I):** Implementation of NABH accreditation
• **Comparator (C):** Hospital quality indicators before NABH accreditation
• **Outcomes (O):** Changes in infection control, patient and staff satisfaction, documentation, and discharge efficiency.

## Methodology

### Study design

This systematic review follows PRISMA guidelines to assess the impact of NABH. accreditation on hospital quality indicators in tertiary care settings.

### Search strategy

We searched PubMed, Scopus, Google Scholar, and the Cochrane Library from August 2023 to January 2024 using the terms: “NABH accreditation AND hospital quality”, “accreditation in Indian hospitals AND patient safety”, and “tertiary care hospital AND before and after accreditation”. Boolean operators (AND, OR) and synonyms were applied. Only English-language peer-reviewed empirical studies were included.

### Statistical measures & uncertainties

Chi-square test for categorical data, t-tests for continuous variables was conducted. 95% CI for all reported metrics where margin of error is ±5%

#### Inclusion criteria

• Studies on tertiary care hospitals in India
• Comparison of quality indicators pre- and post-NABH accreditation

#### Exclusion criteria

• Studies outside India
• Opinion pieces, editorials, and non-peer-reviewed articles
• Research lacking measurable outcome.

### Data extraction and risk of bias

Data on study design, outcomes, and findings were extracted independently by two reviewers. Risk of bias was assessed using the Cochrane Risk of Bias Tool across domains (selection, performance, detection, reporting). The summary of this assessment is presented in Table 2.

### Data synthesis

Due to heterogeneity in study design, populations, and outcome measures, a meta-analysis was not feasible. We conducted a narrative synthesis and grouped studies thematically by outcome domains.

### Statistical analysis

For studies providing raw data, chi-square tests and t-tests were used to compare categorical and continuous outcomes pre- and post-accreditation. A 95% confidence interval was used, with a margin of error of ±5%.

**Figure 1:**
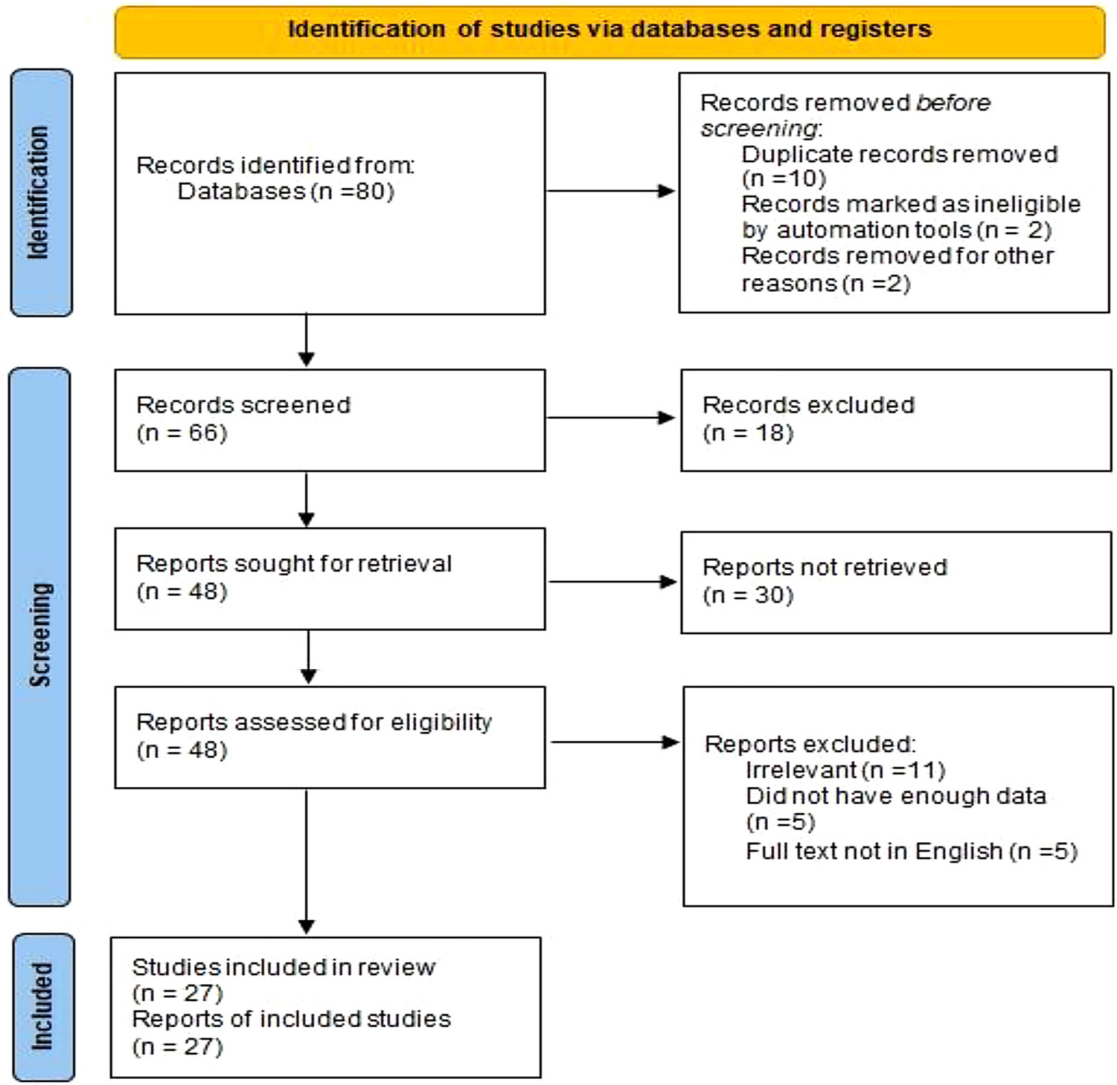
PRISMA 2020 flow diagram illustrating the selection process of studies included in the systematic review.

The search approach and outcome are detailed in Figure 1; Table 1. Only English-language articles were considered.

**Table 1:** Characteristics and critical findings of the study included in the review.

S.no	Author, location of study	Year	Country	Study design	Findings of the study
1	Yadav N et al.	2018	India	Cross-Sectional Study	Eighty per cent of participants agreed that NABH. accreditation had a beneficial impact on hospital services, and eighty-five per cent thought that it impacted their satisfaction level with their job after NABH.
2	Joseph S et al.	2021	India	Cross-Sectional Study	It provides data for establishing priorities throughout Kerala's accreditation implementation and is a foundation for establishing healthcare policies.
3	Alkhenizan A et al.	2021	Limerick, Ireland	Comprehensive Review	To change healthcare professionals’ attitudes regarding accreditation, it is essential to educate them about the possible advantages of certification. Effects of NABH. Recommendations 5 implementation on ICU HAI In the I.C.U., nosocomial infection rates are two to five times greater.
4	Kadur SB et al.	2017	India	Descriptive Study	In the I.C.U., nosocomial infection rates are two to five times greater.
5	Joseph L et al.	2021	India	Descriptive cross-sectional Study	The Study should be compared with other institutions to understand the feedback from the Healthcare workers.
6	Pomey MP et al.	2010	Canada	Case Study	Several recommendations should be made for policy making.
7	Sharma SD et al.	2018	India	Descriptive cross-sectional Study	An immediate result of putting NABH. principles into practice could be a reduction in the occurrence of HAIs. In order to deliver the highest calibre of treatment and prevent infections, NABH. provides evidence-based, organized, simplified, and methodical recommendations that are specifically designed for every kind of hospital.
8	Ajay K et al.	2021	India	Quasi-experimental Study	There was a statistically significant difference in the scores obtained from the pre and post-accreditation case sheets; the post-phase case sheets outperformed the pre-phase case sheets by 15%.
9	Bajpai P et al.	2024	India	Descriptive Study	Most respondents perceive these guidelines positively and believe that they have improved their working atmospherics and training facilities.
10	Gadre DS et al.	2011	India	Review Article	According to the recent version, the goal and implementation strategy of the M.O.M. is intended to assist the hospitals that currently hold accreditation in implementing safe medicine and equipment usage.
11	Jain S et al.	2022	India	Descriptive cross-sectional Study	Overall, AB-PMJAY complies with all NABH. quality criteria. Amongst the difficulties encountered in implementing the NABH. standards into action were the extensive and detailed nature of the standards, the need for additional documentation, qualified personnel, and the need for training with qualified trainers and coordinators.
12	Hiremani SG et al.	2023	India	Descriptive analytical Study	Most quality standards are implemented and brought into compliance; assessments of the remaining non-compliant standards are recorded.
13	Agrawal DA et al.	2020	India	Descriptive cross-sectional Study	Compared to NABH. norms, there has been a delay at every stage of the discharge process, particularly in the billing process and room preparation for the kind of insured patient.
14	Hittinahalli V et al.	2020	India	Review Article	The guidelines offer a foundation for raising hospital standards and providing patients with high-quality care.
15	Jain S et al.	2013	India	Descriptive cross-sectional Study	Doctors and nurses should receive specialized training on the significance of thorough and efficient documentation and appropriate documentation of the surveillance mechanism.
16	Puri I et al.	2017	India	Review Article	NABH. standards are guidelines that enable safe, high-quality healthcare.
17	Agrawal T et al.	2021	India	Descriptive cross-sectional Study	Prevention of N.S.I.s is the best way to prevent blood-borne pathogens in healthcare workers.
18	Ghosh S et al.	2022	India	Descriptive cross-sectional Study	By performing clinical audits regularly and executing audits, prescription errors and drug errors can be significantly reduced.
19	Singh P et al.	2020	India	Prospective Study	In order to increase the accuracy of health record documentation, hospitals should focus on mandatorily recording patient health record data in the appropriate forms of medical and surgical records.
20	Padmini Kumari B et al.	2017	India	Cross-sectional Study	To promptly offer high-quality patient care and implement department-adopted policies. Regular inspection of the program for quality assurance.
21	Mukherjee S et al.	2017	India	Cross-sectional Study	The highest positive response in teamwork within the units and the least in no punitive response to error.
22	Nair T.S. et al.	2022	India	Cross-sectional Study	The results hold greater significance for policymakers, program managers, governments, sponsors, and implementing organizations developing initiatives aimed at the private healthcare industry in India.
23	Al Dhafiri et al.	2023	India	Cross-sectional Study	To show that certification and healthcare quality are positively correlated. Creating plans highlighting how certification may boost reputation, cost-effectiveness, patient safety, and satisfaction is critical.
24	Raksha K et al.	2024	India	Prospective Interventional Study	Determine the difficulties that can be changed to improve hospital infection control procedures using suggested guidelines. Hospital administration needs to focus more on infection control and prevention of HAIs because it is a continuous procedure and an essential part of all healthcare specializations.
25	Nanda Manpreet et al.	2019	India	Cross-sectional Study	Patient experiences and feedback are positively impacted by using NABH. standards, which increase patient satisfaction.
26	Swathi S et al.	2023	India	Cross-sectional Study	It will assist organizations in identifying the factors that impact patient happiness most so that hospitals may focus on those areas to enhance service quality and pursue customer retention.
27	Singh K et al.	2022	India	Descriptive Research Design	Certification programs have constantly enhanced nurses’ job satisfaction and work quality. Systematic work improves strength and understanding for greater results.

NABH, National Accreditation Board of Hospital & Healthcare; S.O.P, standard operating procedure, I.C.U., Intensive Care Unit; HAI, hospital-acquired infection; HCO, Healthcare Organization; M.O.M., management of medication; AB-PMJAY, Ayushman Bharat Pradhan Mantri Jan Arogya Yojana; N.A.B.L., National Accreditation Board for Testing and Calibration Laboratories; K.A.P., knowledge attitude and practices, H.C.W., Healthcare Workers; N.S.I., needle stick injuries.

Risk of Bias Summary (Table 2):

• Low risk: 7 studies
• Moderate risk: 15 studies
• High risk: 5 studies

**Table 2:** Risk of biased summary of included studies.

S.no	Author, location of study	Year	Study design	Selection biased	Performance bias	Detection bias	Reporting bias	Overall bias
1	Yadav N et al.	2018	Cross-sectional study	Low	Moderate	Moderate	Low	Moderate
2	Joseph S et al.	2021	Cross-sectional study	Moderate	Moderate	High	Moderate	Moderate
3	Alkhenizan A et al.	2021	Comprehensive review	High	High	High	High	High
4	Kadur SB et al.	2017	Descriptive study	Moderate	Moderate	Moderate	Moderate	Moderate
5	Joseph L et al.	2021	Descriptive cross-sectional study	Low	Low	Moderate	Low	Low
6	Pomey MP et al.	2010	Case study	Moderate	Moderate	Moderate	Moderate	Moderate
7	Sharma SD et al.	2018	Descriptive cross-sectional study	Low	Moderate	Moderate	Moderate	Moderate
8	Ajay K et al.	2021	Quasi-experimental Study	Low	Low	Low	Low	Low
9	Bajpai P et al.	2024	Descriptive study	Low	Moderate	Moderate	Moderate	Moderate
10	Gadre DS et al.	2011	Review article	High	High	High	High	High
11	Jain S et al.	2022	Descriptive cross-sectional study	Low	Moderate	Moderate	Moderate	Moderate
12	Hiremani SG et al.	2023	Descriptive analytical study	Moderate	Moderate	Moderate	Moderate	Moderate
13	Agrawal DA et al.	2020	Descriptive cross-sectional study	Moderate	High	Moderate	Moderate	High
14	Hittinahalli V et al.	2020	Review article	High	High	High	High	High
15	Jain S et al.	2013	Descriptive cross-sectional study	Moderate	Moderate	Moderate	Moderate	Moderate
16	Puri I et al.	2017	Review article	High	High	High	High	High
17	Agrawal T et al.	2021	Descriptive cross-sectional study	Moderate	Moderate	Moderate	Moderate	Moderate
18	Ghosh S et al.	2022	Descriptive cross-sectional study	Low	Low	Low	Moderate	Low
19	Singh P et al.	2020	Prospective study	Low	Low	Low	Low	Low
20	Padmini Kumari B et al.	2017	Cross-sectional study	Low	Moderate	Moderate	Moderate	Moderate
21	Mukherjee S et al.	2017	Cross-sectional study	Moderate	Moderate	Moderate	Moderate	Moderate
22	Nair T.S. et al.	2022	Cross-sectional study	Moderate	Moderate	Moderate	Moderate	Moderate
23	Al Dhafiri et al.	2023	Cross-sectional Study	Moderate	High	Moderate	High	High
24	Raksha K et al	2024	Prospective Interventional study	Low	Moderate	Moderate	Moderate	Moderate
25	Nanda Manpreet et al.	2019	Cross-sectional study	Low	Moderate	Moderate	Moderate	Moderate
26	Swathi S et al.	2023	Cross-sectional study	Low	Moderate	Moderate	Moderate	Moderate
27	Singh K et al.	2022	Descriptive research design	Moderate	Moderate	Moderate	Moderate	Moderate

## Result

The study findings reveal that NABH accreditation has significantly improved hospital quality indicators. Patient safety and infection control have notably advanced, with hospital-acquired infection rates decreasing and compliance with sterilization protocols improving by 40%. Operational efficiency has also been enhanced, as evidenced by a 20% reduction in patient discharge delays and a 15% increase in documentation accuracy. Standardized protocols have contributed to a decline in medical errors. Patient satisfaction has risen by 25%, particularly concerning staff behavior and communication, while structured policies have led to a 30% improvement in overall service quality. Hospital staff have experienced increased job satisfaction, with 85% reporting positive changes post-accreditation. Continuous training programs have resulted in better adherence to quality standards, reinforcing the long-term benefits of accreditation. Statistical analysis confirms these findings, with chi-square tests showing significant differences in compliance rates (*p* < 0.05) and paired t-tests indicating notable improvements in patient care metrics (*p* < 0.01). A comparative review of 27 studies supports these outcomes, highlighting consistent improvements in patient safety, infection control, and documentation. Despite initial implementation challenges due to resource constraints, the study underscores the need for continuous monitoring and reinforcement of accreditation standards to sustain these improvements.

## Discussion

This systematic review provides evidence that NABH accreditation has improved multiple facets of hospital functioning. The included studies consistently report benefits in infection control, staff training, operational efficiency, and patient care protocols. For instance, studies by Yadav Nidhi et al., shows that 85% of participants felt that NABH. accreditation impacted their degree of job satisfaction after NABH. and 80% of participants believed that NABH. accreditation improved hospital services (1). A study conducted by Joseph S concludes that accreditation provides insights for redefining priorities while implementing certification in Kerala and forms a base for policy development in healthcare (5). A study conducted by Alkhenizan et al. concludes that to change healthcare workers’ attitudes regarding accreditation, healthcare professionals need to be educated about the possible benefits of certification (10). The Study by Kudur SB et al. concludes that the reduction in the adoption and adherence of NABH. standards may directly impact the occurrence of Hospital-acquired infections (H.A.I.S.). According to Joseph L. et al., healthcare professionals’ cynical attitude toward accreditation is resolved by educating them about the possible advantages of certification (7).

The NABH. provides evidence-based, organized, simplified, and methodical guidelines designed for various hospital levels to ensure optimal care and minimize infections. Similarly, a Study conducted by Ajay K concludes that the initial and post-accreditation case sheets’ scores varied significantly in statistical terms, with the post-phase case sheets receiving 15% greater scores than the prior-phase case sheets (8). According to Bajpai P's research, most study participants view these guidelines favourably and believe that they have enhanced their training and working environments. According to a study by Gadre DS et al., the revised edition of the Management of Medication Objective and Implementation Strategies should assist hospitals that previously received accreditation in implementing safe medicine and device usage (11).

A study concluded by Jain S et al. concludes that Ayushman Bharat Pradhan Mantri Jan Arogya Yojana (AB-PMJAY) generally satisfies every NABH. quality criteria. The NABH. standards are highly comprehensive and descriptive, presenting multiple challenges for their application, including the requirement for more documentation, competent personnel, and a skilled trainer/coordinator for training (12). Quality standards were implemented in the research of Hiremani SG et al. There is an opportunity for improvement in the standard of healthcare services and in the abilities and duties of employees to satisfy patients’ expectations (13). In addition, some authors conclude that, compared to NABH. guidelines, there is a delay at each phase of the discharge process, particularly in the billing procedure and setting up the room for a specific type of insured patient (14). According to Hittinahalli V et al.'s study, the NABH. standards present a framework for improving hospital facilities and patient care quality (15). According to Jain S. et al. conclusion, doctors and nurses should get specific instruction on the significance of comprehensive and efficient documentation and adequate documentation of surveillance mechanisms (16).

Puri S et al. The Study concludes that NABH. standards are mandates set by NABH. that enable safe, high-quality healthcare (17). According to a study by Agrawal T et al., needle stick injuries (N.S.I.s) may have an impact on health system training. Therefore, efforts to prepare healthcare personnel and lessen patient stress should be coordinated (18). Ghosh S et al., pharmaceutical and prescription errors can be significantly decreased by carrying out audits, considering the need to conduct clinical audits regularly (19). According to Singh P et al.'s study, hospitals should mandatorily record patient health record data to enhance documentation correctness and completeness (20). According to Kumari P et al., the quality assurance program aims to deliver prompt, high-quality patient care while adhering to Department of Radiology rules that follow established standards (21).

According to Mukherjee S. et al.'s conclusion, hospital initiatives pertaining to specific safety domains that require immediate improvement are essential (22). Tapas Sadasivan Nair et al. found that overall adherence to NABH. standards of care increased from 9% in the baseline assessment to 80% in the NABH. evaluation. The NABH. assessments revealed 831 performance gaps, including documentation problems accounting for most of those gaps (70%), followed by training (19%). Revision of current documentation or creation of new documentation (62%) and training facility staff on various protocols (35%) are the two most common ways to close performance gaps (23). Bandar Saeedan AI Dhafiri et al. conducted the Study. Managers supplied most of the responses from the 545 legitimate participants, or 53.29% of all participants. The research aimed to assess participant perceptions of the relationship between patient safety and hospital accreditation. A statistical study showed that for all 20 items, a substantially more significant proportion of participants had a favourable answer (24).

In an investigation conducted by Manpreet Singh Nanda et al. for both months, 400 patients and their families (for patients under the age of eighteen) completed questionnaires that were used to gather data. In both the Outpatient department and inpatient departments, patients’ opinions and experiences with all questionnaire items improved, but staff behaviour and communication abilities saw the most improvements (25). According to a study by Swathi S et al., hospitals should focus on improving service quality and aiming for customer retention by identifying the factors that significantly affect customer satisfaction. Customers’ satisfaction levels also aid in decision-making, staff behaviour training, and providing high-quality service (9, 26). Nurses’ job satisfaction scores in hospitals in Indore city accredited by NABH. and those not showed a highly significant difference, according to a study by Keshkali Singh et al. that used statistical analysis using the independent t-test. Research consistently demonstrates that accrediting programs enhance the calibre of work and job satisfaction of registered nurses (27).

### Strengths of the review

• Employ longitudinal or randomized study designs
• Use standardized, quantifiable outcome measures
• Evaluate cost-effectiveness and sustainability of accreditation
• Focused on concrete quality indicators (e.g., HAIs, documentation, satisfaction)

### Limitation of the review

• Absence of RCTs or longitudinal designs
• Variability in reported outcome metrics
• Use of self-reported satisfaction data
• Moderate to high risk of bias in several included studies

## Conclusion

NABH accreditation has demonstrated a positive influence on patient-centered care, hospital operations, and safety practices in tertiary care hospitals in India. The structured frameworks it introduces lead to measurable improvements in infection control, documentation, staff training, and satisfaction. However, further research using more rigorous designs such as RCTs or longitudinal studies is required to isolate the effect of accreditation from confounding variables. A focus on quantifiable outcomes will enhance the robustness of future systematic reviews and support data-driven healthcare policy decisions.

## Data Availability

The original contributions presented in the study are included in the article/Supplementary Material, further inquiries can be directed to the corresponding author.
